# Molecular Strategies of Carbohydrate Binding to Intrinsically Disordered Regions in Bacterial Transcription Factors

**DOI:** 10.3390/ijms27020941

**Published:** 2026-01-17

**Authors:** Yuri A. Purtov, Olga N. Ozoline

**Affiliations:** Department of Functional Genomics of Prokaryotes, Institute of Cell Biophysics of the Russian Academy of Sciences, Federal Research Center Pushchino Scientific Center for Biological Research of the Russian Academy of Sciences, 142290 Pushchino, Russia

**Keywords:** bacterial transcription factors, intrinsically disordered regions, flexible inter-domain linkers, effector-binding pockets, metabolic carbohydrates, sequential molecular docking

## Abstract

Intrinsically disordered regions enable transcription factors (TFs) to undergo structural changes upon ligand binding, facilitating the transduction of environmental signals into gene expression. In this study, we applied molecular modeling methods to explore the hypothesis that unstructured inter-domain and subdomain linkers in bacterial TFs can function as sensors for carbohydrate signaling molecules. We combined molecular dynamics simulations and carbohydrate docking to analyze six repressors with GntR-type DNA-binding domains, including UxuR, GntR and FarR from *Escherichia coli*, as well as AraR, NagR and YydK from *Bacillus subtilis*. Protein models obtained from different time points of the dynamic simulations were subjected to sequential carbohydrate docking. We found that the inter-domain linker of the UxuR monomer binds D-fructuronate, D-galacturonate, D-glucose, and D-glucuronate with an affinity comparable to nonspecific interactions. However, these ligands formed multimolecular clusters, a feature absent in the UxuR dimer, suggesting that protein dimerization may depend on linker occupancy by cellular carbohydrates. D-glucose interacted with linkers connecting subdomains of the LacI/GalR-type E-domains in GntR and AraR, forming hydrogen bonds that connected distant structural modules of the proteins, while in NagR, FarR and YydK, it bridged the inter-domain linkers and a β-sheet within the HutC-type E-domains. Hence, our results establish flexible linkers as pivotal metabolic sensors that directly integrate nutritional cues to alter gene expression in bacteria.

## 1. Introduction

Although bacteria possess diverse adaptive regulatory strategies, transcription is the primary control point for modulating gene expression [1,2]. This process is orchestrated by transcription factors (TFs), whose evolutionarily optimized structural domains enable a specific response to environmental signals. Nearly 42% of TFs are single-domain proteins [3], built from a limited repertoire of structural modules specialized for DNA sequence recognition at their binding sites [4,5]. Most TFs (50.2%) contain a second domain that interacts with RNA polymerase (RNAP) or other transcriptional machinery components, while the remaining ~8% are organized into three or more domains with distinct or overlapping functions [3]. Beyond these structured domains, TFs often contain flexible inter-domain linkers or unstructured terminal regions [6]. These dynamic segments are critical for allosteric regulation, modulation of DNA and RNA polymerase (RNAP) binding, and signal transduction. For example, in *Escherichia coli* (*E. coli*), the AraC protein represses transcription of the araBAD operon in the absence of arabinose. Arabinose binding to its effector domain triggers a conformational shift in the AraC linker from an α helix to a flexible state, enabling the dimeric DNA-binding domains to engage the araBAD promoter and activate transcription [7]. Conversely, maltotriose binding to the effector domain of the TF MalT induces structural ordering of its unstructured N-terminal loop, facilitating MalT oligomerization and subsequent transcription activation [8]. Despite several examples, it is currently unclear to what extent TFs’ regulatory ability depends on the inherent plasticity of their disordered segments.

The functional significance of unstructured protein segments has become widely recognized over the past two decades [9,10,11]. This has led to the formulation of the “disorder–function paradigm” complementing the classical “structure–function paradigm” that equates protein function with well-defined three-dimensional structures [10]. Intrinsically disordered proteins (IDPs) and intrinsically disordered regions of proteins (IDRs) are now considered essential components in a wide variety of cellular processes [12]. Lacking stable secondary and tertiary structures, IDPs/IDRs function as highly plastic molecules that complement the role of globular proteins in cells [13,14]. A central question that has persisted since their discovery is how these proteins recognize their targets and perform specific functions despite their structural heterogeneity [15,16,17]. Since the report on local structural elements such as a pre-structured helix and two turns in the target-unbound state of the p53 transactivation domain [11], transient secondary structures found in dozens of unbound IDPs/IDRs [17] have provided the most reliable answer to this question. Available genomic data indicate a striking difference in the abundance of proteins with unstructured modules across the three domains of life. Whereas only 2.0–4.2% of prokaryotic proteins contain disordered regions longer than 30 residues, this proportion rises to 33% in eukaryotes [18]. However, Molecular Recognition Elements/Features (MoRE/Fs), defined as disordered segments that undergo binding-induced folding [19,20,21], are present in 29% of prokaryotic IDRs and 21% of eukaryotic IDRs [20]. This comparable ratio suggests a roughly equal propensity for disorder-to-order transitions in prokaryotes and eukaryotes and is especially relevant given that bacterial transcription factors and other DNA-binding proteins are significantly enriched among proteins containing MoREs.

IDRs most often exist as localized segments within a protein [13,14], frequently as flexible linkers between globular domains [22] or as unstructured protein termini [6,23]. When positioned between domains, these linkers not only control domain orientation but also facilitate interactions with small molecules [24]. The structural outcomes of these interactions are diverse, spanning a continuum from rigid, ordered complexes to highly unconstrained states with multiple weak contacts [16,25,26,27]. A key functional consequence is allosteric regulation. It is well-established that IDRs significantly contribute to allosteric modulation in multi-domain proteins, and they can do so without folding into secondary structure elements [14,23,28,29]. By acting solely as flexible linkers that interact with ligands, other linkers or globular domains of the same or other proteins, IDRs can trigger domain reorientation or induce conformational changes within them [30,31,32]. Therefore, flexible polypeptide chains are not mere structural connectors [33,34].

The Dynamic Allostery model posits that flexibility of unstructured modules in proteins provides ensembles of conformations that can be sampled [34]. Transitions between these states proceed “along major allosteric propagation pathways” that are pre-encoded within the linker sequences. This process defines a series of sequential transitions between preferred states, where each preceding state determines the subsequent one. Functionally significant linker conformations may represent rare states in the inactive protein that become more populated due to allosteric “maturation”. For IDRs, this directed folding upon ligand binding could stabilize specific conformations [11,35]. Such stabilization can occur via “conformational selection”, where a pre-existing state is stabilized [36], or through “induced fit”, where ligand binding induces and stabilizes a new conformation [37,38]. Ultimately, binding specificity is thought to arise from distinct frustration patterns encoded within the heterogeneous conformational landscape of the binding region [39].

Allosteric communications between different parts of a protein provide an efficient way to turn protein function on or off in response to cellular signals. This is particularly important for regulatory proteins, like transcription factors [11,40,41] and signal transduction proteins [42,43,44]. However, recent studies suggest that the regulation of a protein’s function at one site by a change at a distant site is an intrinsic property of virtually all dynamic proteins, moving beyond the classical view that it is exclusive to specialized “allosteric” proteins [45,46]. Decades of research have established fundamental principles for how regulatory signals are perceived and transmitted. It is now widely acknowledged that ligand binding induces local conformational changes that promote a dynamic sampling of the protein’s conformational landscape. This process selects the most stable conformers from a preexisting ensemble [47,48,49,50]. Allosteric signals are then transmitted through the formation of transient hydrogen bonds [51,52] that propagate along multiple, pre-existing pathways [53], which can be altered by mutations [54]. It is likely that this transmission can occur even without global alterations in the protein backbone [53,55,56]. Ligand binding thus increases the population of stabilized conformers while preserving the protein’s ability to bind other ligands [57]. This mechanism, known as “conformational selection and population shift”, suggests that any ligand can shift the equilibrium of the dynamic conformational ensemble, thereby inducing an allosteric response via evolutionarily tuned dynamic trajectories.

Intrinsically Disordered Proteins (IDPs) are a distinct category of proteins that lack a stable tertiary structure entirely. Although less common than proteins containing IDRs, IDPs constitute an average of 1.6% of bacterial proteomes [58]. Structural fluctuations and conformational heterogeneity in IDPs, revealed by NMR spectroscopy [11], support the hypothesis that IDPs exist as dynamic conformational assemblies stabilized by transient intramolecular interactions [23,59]. Their primary sequences are grouped into modular elements such as short linear motifs (SLiMs), low-complexity domains (LCDs) and MoRFs [60]. Consequently, several IDPs have been identified among eukaryotic transcriptional regulators. A key examples is the human transcription-activating protein HMGN5 (ID P82970), which is fully unstructured [61,62]. HMGN5 counteracts chromatin compaction by binding to nucleosomes [63] and can also interact with cellular RNAs. Its binding site for snoRNA2T2 partially overlaps with its nucleosome-binding site [64]. Furthermore, the transcription factor CTCF (CCCTC-binding factor) has been identified as an in vivo interaction partner of HMGN5 [64]. These findings illustrate the remarkable functional versatility of a completely unstructured regulator, whose conformational flexibility enables interactions with diverse partners. Such binding promiscuity is a hallmark of IDPs. This adaptability is so pronounced that even very short (34–40 residues) unstructured fragments from proteins like PUMA (an apoptosis regulator protein) and E1A (the adenovirus hub protein) undergo conformational changes in response to small molecules like glycine, tryptophan, and sarcosine [65]. This suggests that fluctuations in the intracellular solute composition, which commonly occur within cells, can directly modulate the function of proteins with intrinsically disordered regions.

Most TFs respond to environmental signals through allosteric regulation, typically triggered by effector molecules such as metabolites from the very pathways they control [66]. Effector binding usually induces conformational changes that either modulate DNA-binding affinity or alter protein–protein interaction interfaces. One of the largest and most widespread TF families in prokaryotes is the GntR-type regulator family [67,68,69]. Found across diverse bacterial lineages, these proteins regulate various cellular processes, including carbon metabolism, antibiotic production, and the cell wall stress response. Structurally, proteins from the GntR family consist of two primary domains: an N-terminal DNA-binding domain and a C-terminal effector-binding/oligomerization (E-O) domain, connected by flexible linkers [69]. While all proteins from the GntR family share a highly similar structural organization in their N-terminal DNA-binding domains [67], they are classified into four major (FadR, HutC, MocR, YtrA) and two minor subfamilies (AraR and PlmA) based on the structural variations in their C-terminal domains [67,70,71]. Ultimately, effector binding triggers allosteric signals that propagate through oligomerization or conformational changes within the E-O domains [54,67,72,73,74].

While unstructured inter-domain linkers are no longer considered passive connectors, their role in effector binding and intra-domain dynamics remains unclear. Although some ligands can stabilize these flexible linkers in specific conformations, traditional structural biology methods can only capture a limited number of these conformers. Given the vast conformational diversity of intrinsically disordered regions, their potential role in perceiving metabolic signals should not be neglected. Therefore, in this study, we evaluate the ability of the intrinsically disordered regions in transcription factors from the GntR family to interact with key substrates and intermediates from central bacterial metabolic pathways. We employed molecular dynamics (MD) simulations [75] to sample conformers of the apo-proteins from various time points of the dynamic trajectories. These conformers were then subjected to flexible molecular docking [76] to estimate the availability of the unstructured protein regions for carbohydrates. Performing molecular docking sequentially for ten molecules of each carbohydrate type allowed us to quantify the background level of protein-carbohydrate interactions, sugar preference for unstructured protein chains, and linker occupancy. Finally, we used the Protein-Ligand Interaction Profiler (PLIP) [77] to visualize the hydrogen bonds potentially formed by the carbohydrates that exhibited the highest affinity for the unstructured regions.

## 2. Results

### 2.1. Modes of Carbohydrate Interaction with Inter-Domain Linkers of the E. coli UxuR TF

The UxuR repressor controls the transcription of genes encoding key enzymes of the Ashwell and Entner–Doudoroff pathways, which integrate hexuronates and hexuronic acids into the glycolytic cycle [78,79]. The protein functions as a homo- or heterodimer with its paralog, ExuR [79,80], and represses the transcription of its own gene as well as several other operons involved in the transport and degradation of sugar acids [66]. Although the crystal structure of UxuR is not yet available, homology modeling and molecular docking have identified a zinc-binding motif within the C-terminal domain [81]. In the presence of fructuronate or its isomer glucuronate, the transcription of *uxuR* is induced, and it is hypothesized that this induction is mediated by a conformational transition in UxuR that increases the negative charge on the surface of its N-terminal domains [81]. In addition, UxuR is subject to catabolic regulation by D-glucose and cyclic AMP [82]. Given this complex regulation, the structural impact of potential carbon effectors has been studied in silico using MD simulation and sequential molecular docking [73,80,83]. In our previous study [73], we identified two predominant sugar-binding sites on the UxuR monomer using eleven small intermediates from the Ashwell and Entner–Doudoroff pathways. The first site, located between the N- and C-terminal domains, exhibited a roughly similar affinity for all tested sugars. The second site, found within the effector-binding domain (E-domain), had the highest affinity for D-fructuronate and D-glucose [73]. In a subsequent study [83], we employed an additional set of natural carbohydrates to evaluate steric constraints imposed by the conformations of the inter-domain linkers in the UxuR dimer and the size of the protein pocket in the C-terminal domains. This set included small molecules, such as glucose-6-phosphate and fructose-6-phosphate, as well as more complex sugars like lacto-N-fucopentaose, lacto-N-difucohexaose, and lacto-N-tetraose. As expected, not all complex carbohydrates could enter the CTD pocket. However, the inter-domain linkers interacted with all tested sugars. The main objective of the present study is to evaluate the occupancy of these linkers by the natural effectors of UxuR using conformers generated at different time points from molecular dynamic simulations.

#### 2.1.1. The Flexible Inter-Domain Linker of the UxuR Monomer Exhibited Low Carbohydrate Binding Efficiency but Provided a Platform for Their Clustering

**Figure 1:**
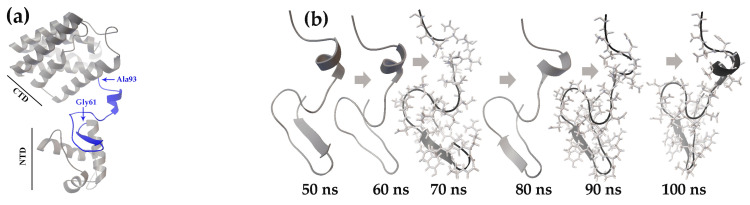

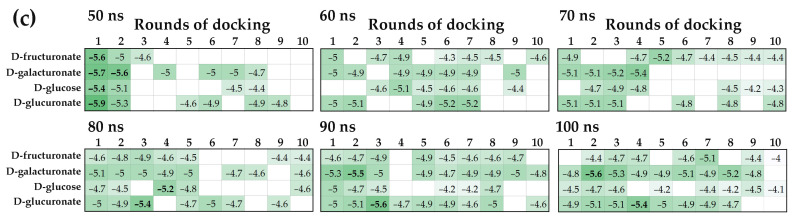
MD simulations revealed conformational flexibility of the *E. coli* UxuR inter-domain linker, which affected carbohydrate-binding affinity. (**a**) Monomer structure of the UxuR predicted by the SWISS-MODEL. The region spanning from Gly61 to Gly94 is highlighted in blue. (**b**) Sequential rearrangements of the UxuR inter-domain linker at 50–100 nanoseconds of the MD trajectory. The linker from the 100 ns model and linkers lacking an α-helix are shown with amino acid side chains. (**c**) Heat maps representing the affinity of carbohydrate interactions with the UxuR monomer’s inter-domain linker (ΔG kcal/mol). White cells correspond to random, nonspecific complexes on other protein surfaces, which had average ΔG values of −4.52 ± 0.26 (D-fructuronate), −4.85 ± 0.20 (D-galacturonate), −4.45 ± 0.23 (D-glucose) and −4.82 ± 0.18 (D-glucuronate) kcal/mol. ΔG values greater than 3 StDs above the mean nonspecific binding are shown in bold.

Figure 1a shows the structure of the UxuR monomer, predicted by the SWISS-MODEL online server [84,85,86]. It comprises a canonical GntR family N-terminal DNA-binding domain with a winged helix-turn-helix (wHTH) motif and a bulky “all helical” C-terminal FadR-type [70] domain predicted for effector binding. The protein chain between the DNA-binding domain and CTD (L74-G94) is unstructured in the UxuR UniProt model P39161 [87]. However, our MD simulations showed the ability of its amino acid sequence (Gly61–Gly94) to form short transient alpha-helices, and the double-stranded beta-sheet at the exit of the wHTH module was unstable (Figure 1b). Thus, it is likely that the UxuR inter-domain linker may be one of those IDRs that are able of forming pre-structured target-binding motifs [17,21,36,88]. 

The results of sequential molecular docking are summarized in Figure 1c. They indicate that the inter-domain linker of the UxuR protein interacted with models of every selected carbohydrate type at all time points sampled from the MD trajectory. Although the binding affinity in most cases did not exceed the average level of random surface binding by more than three standard deviations (StDs), at least three molecules of each carbohydrate were consistently positioned in the inter-domain linker region. The highest binding affinity, which was statistically significant against the background, was observed for ligands bound to the linker region of the 50 ns model in the first round of docking. However, the average occupancy of the linker progressively increased in models from later simulation times. Specifically, the linker in the 90 ns UxuR model was associated with 6–9 sugar molecules, while the 100 ns model interacted with 8–9 carbohydrates (Figure 1c). Thus, despite the low binding affinity of most sugars to the linker region, which is typical for nonspecific interactions, we observed a clear preferential occupancy of this region by multiple molecules, which, on the contrary, is characteristic of specific binding.

**Figure 2:**
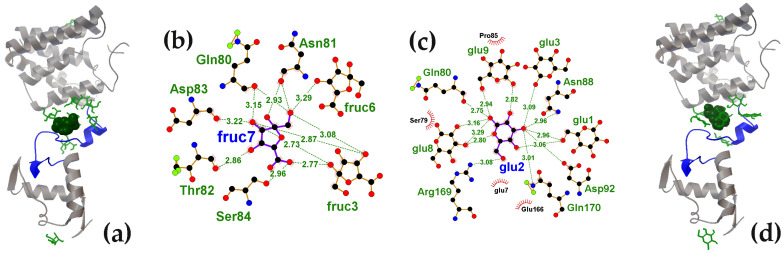
Preferential binding and cluster formation of D-fructuronate and D-glucose in the UxuR monomer linker region, potentially stabilized by hydrogen bonds between ligand molecules. (**a**,**d**) The UxuR models (ribbon) from a 100 ns MD trajectory with bound D-fructuronate and D-glucose molecules (green rods). The ligands with the highest binding affinity are shown as ball models. (**b**,**c**) LigPlot+ diagrams (generated with PLIP v.2.2.8) depicting hydrogen-bond interactions for D-fructuronate (from the 7th round of molecular docking) and for D-glucose (from the 2nd round).

Figure 2 shows the surface distribution of D-fructuronate and D-glucose on the UxuR monomer. D-fructuronate relieves UxuR-mediated repression [81], whereas D-glucose integrates UxuR into catabolite repression networks [82]. All ten molecules of each ligand sequentially used in molecular docking are visualized on a 100 ns snapshot from UxuR molecular dynamic simulations. Ligands contacting the linker region formed clusters (Figure 2a,d), which are likely stabilized by hydrogen bonds, predicted by Protein-Ligand Interaction Profiler [77] (Figure 2b,c). While the carbohydrate clusters of D-fructuronate and D-glucose were of similar size (7 and 8 molecules, respectively; Figure 1c), their topologies within the protein’s inter-domain space differed (Figure 2a,d). In particular, the D-fructuronate molecule with the highest affinity for UxuR (ball model in Figure 2a) was within hydrogen bond (H-bond) distance of Gln80, Asn81, Thr82, Asp83 and Ser84 (Figure 2b), whereas the highest-affinity D-glucose molecule, based on its predicted location, could form H-bonds with Gln80, Asn88, Asp92, Arg169 and Gln170. Moreover, within these clusters H-bonds can form not only between the ligand and the protein but also between ligand molecules. For instance, the interaction of D-fructuronate with UxuR in the 7th docking round may be stabilized by H-bonds formed with D-fructuronates attached to the protein in the 3rd (fruc3) and 6th (fruc6) rounds (Figure 2b). Similarly, in the glucose binding site, glu2 appears to be stabilized by glu1, which may then facilitate the binding of glu3, glu8 and glu9 (Figure 2c).

**Figure 3:**
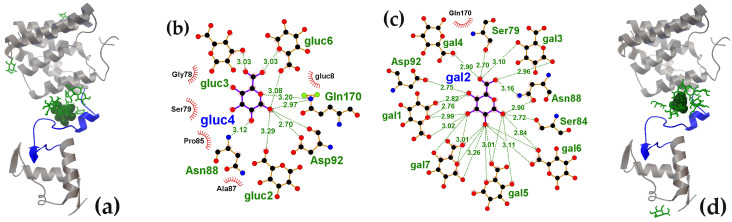
Preferential binding and cluster formation of D-glucuronate and D-galacturonate in the UxuR monomer linker region, potentially stabilized by H-bonds formed between ligand molecules. (**a**,**d**) The UxuR model from a 100 ns MD trajectory with bound D-glucuronate and D-galacturunate molecules (green rods). The ligand with the highest binding affinity is shown in ball model. (**b**,**c**): LigPlot+ diagrams predicting the hydrogen bond interactions for D-glucuronate from the 4th round of molecular docking (**b**) and for D-galacturunate from the 2nd round.

Eight molecules of D-glucuronate (an isomer of D-fructuronate), were found to occupy a nearly identical region in the UxuR inter-domain space (Figure 3a). Among these, the molecules docked in the second (gluc2), third (gluc3) and the sixth (gluc6) rounds (Figure 1c) could form H-bonds with the highest-affinity D-glucuronate molecule (gluc4) (Figure 3b). In the case of D-galacturonate (a stereoisomer of D-glucuronate), the network of potential H-bonds was the most diverse (Figure 3c). While Asn88 and Asp92 interacted with both stereoisomers, the D-galacturonate molecule from the second docking round could also form H-bonds with Ser79, Ser84 and six other D-galacturonate molecules (Figure 3c,d).

Thus, although the interactions of D-glucose and the three hexuronate isomers with the UxuR inter-domain linker occurred at near-background efficiency levels, molecular docking revealed some binding preferences and showed that ligand-ligand interactions can stabilize complex formation. Since sequential docking to some extent simulates a gradually increasing ligand concentration, the observed clustering suggests that similar structuring in vivo could create aggregates that restrict the linker’s conformational mobility. This would thereby stabilize it in a functionally significant state or, conversely, limit its intermolecular interactions.

#### 2.1.2. Carbohydrate Interactions with the UxuR Dimer: No Cluster Formation in Inter-Domain Linkers but Connection to the α-Helix of the Effector Domain

**Figure 4:**
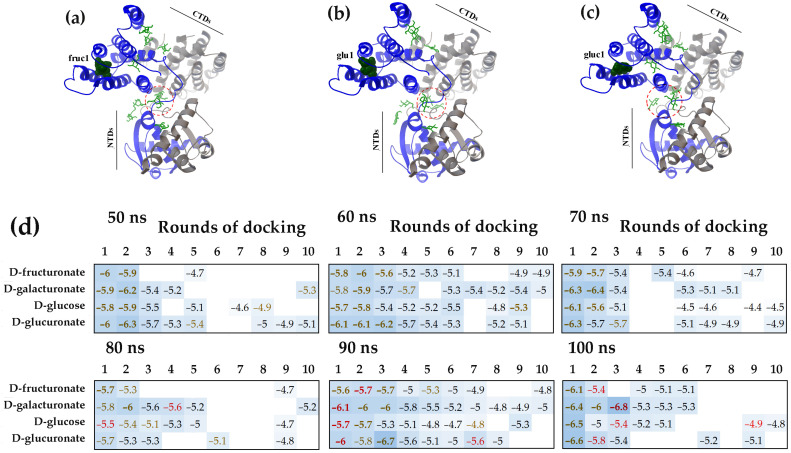
Confirming the previously observed interaction of carbohydrates with the UxuR effector-binding domain [73,83], molecular docking revealed a novel binding mode, in which ligands connect linkers with CTD. (**a**–**c**) Dimer structures of UxuR (ribbon) from a 100 ns MD trajectory with bound D-fructruronate (**a**), D-glucose (**b**) and D-glucuronate (**c**) molecules (green rods). The ligand with the highest binding affinity in each complex is shown as a ball model. Red circles outline sugars that form contacts with both the linker and the CTD α-helix. (**d**) Heat maps representing the binding affinity (ΔG kcal/mol) of hexuronates for the UxuR dimer over successive molecular docking rounds. ΔG values are categorized by binding site: CTD pocket (brown), inter-domain linkers only (black), and linkers connected by sugar ligand to the CTD (red). White cells correspond to nonspecific complexes formed on other protein surfaces, which had average ΔG values of −4.88 ± 0.24 (D-fructuronate), −5.26 ± 0.21 (D-galacturonate), −4.74 ± 0.26 (D-glucose) and −5.22 ± 0.22 (D-glucuronate) kcal/mol. ΔG values corresponding to statistically significant binding are bolded.

In the UxuR dimer, the inter-domain linkers are located in close proximity to each other (Figure 4a–c), creating a spatial constraint that limits ligand access to these regions. Consequently, a greater number of carbohydrates bound to other dimer surfaces (Figure 4d) compared to the monomer (Figure 1c). A primary binding site was the region where the alpha-helices of the C-terminal domain converge, which corresponds to the entrance of the previously identified carbohydrate-binding pocket [73,83]. This site predominantly bound ligands in the first rounds of molecular docking and typically exhibited the highest affinities of binding (indicated by brown ΔG values in Figure 4d). Such interaction pattern was observed across protein models derived from various time points of the MD trajectory and was most pronounced for the complexes formed with the 100 ns UxuR model.

**Figure 5:**
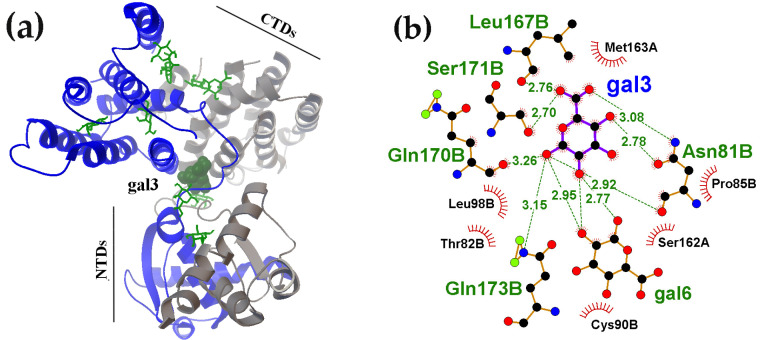
Carbohydrates can form intramolecular bridges connecting the inter-domain linkers to the UxuR CTD. (**a**) Structures of the UxuR dimer from a 100 ns MD trajectory, showing bound D-galacturonate molecules. The ligand bridging Asn81 in the linker to residues Leu167, Gln170, Ser171 and Gln173 on the CTD α-helix is shown as a ball model. (**b**) Correspondng LigPlot+ diagram visualizing predicted H-bond interctions, with bond distances provided in Ångströms. The amino acid residues are indicated as belonging to monomers A and B of the UxuR dimer.

Although fewer ligands occupied the inter-domain linkers in the UxuR dimer than in its monomer, their average binding affinity for all tested carbohydrates was slightly higher (Figure 2d and Figure 4d). Moreover, several ligands interacting with the 90 ns and 100 ns models of the UxuR dimer exhibited affinities greater than those bound to the potential effector-binding site (shown by the red and brown ΔG values in Figure 4d, respectively). D-galacturonate demonstrated the highest affinity when it bridged the inter-domain linker of the 100 ns model at the third round of docking with the CTD (Figure 4d and Figure 5). Notable, all ligands bound to this specific site are able to form H-bonds with amino acid residues from both the inter-domain linker and the C-terminal domain of the protein (Figure 5a,b). This represents a new mode of carbohydrate interaction with the flexible linkers of UxuR. The observation that such interaction occurred exclusively in protein models from the late stages of the MD trajectory suggests that the competent binding site was not formed spontaneously. Instead, its formation likely required a sequential conformational selection, a process the simulation successfully captured for all ligands.

The limited space at the dimer’s inter-domain linker interface likely explains the absence of active carbohydrates clustering in this region. Nevertheless, the ability of ligands from different molecular docking rounds to form hydrogen bonds with each other was detected. Figure 5b, in particular, shows the arrangement of two D-galacturonate molecules, where the gal3 bridges the linker and the CTD α-helix while also facilitating the attachment of a second D-galacturonate molecule (gal6) to the linker region. Thus, the functional repertoire of inter-domain linkers may extend beyond adopting specific conformations depending on the bound partner. Multi-molecular ligand clustering could potentially influence dimerization dynamics, while intramolecular bridging could modulate relative orientation of the domains. Although interactions with the linkers generally exhibited low affinity, the spatial proximity of Asn81 to Leu167, Gln170, Ser171 and Gln173 in the UxuR α-helix could enable not only strong but also specific carbohydrate binding, at least for D-galacturonate (Figure 5b).

### 2.2. Similarities and Diversity of D-Glucose Binding Modes with Unstructured Linkers of Other Transcription Factors

In this phase of the study, we investigated whether unstructured linkers from other TFs could participate in potentially functional interactions with carbohydrates. We selected five proteins for modeling as outlined in the Materials and Methods. The selection included two TFs from *E. coli* (GntR and FarR) and three from *Bacillus subtilis* (*B. subtilis*) (AraR, NagR, and YadK). None of the tested carbohydrates is a specific effectors for these TFs. However, existing data suggest that the effector-binding sites of many TFs can accommodate small molecules from even different metabolic pathways [89], a feature we anticipated would be more pronounced in IDRs. Because our data on UxuR-ligand complexes showed no statistically significant difference in the interaction with different sugars, we used only D-glucose as a universal probe for linker accessibility. All five selected proteins function as dimers, but we evaluated ligand interactions only with monomeric forms to minimize the likelihood of the linkers being shielded at the dimer interface, as was observed for the UxuR dimer.

#### 2.2.1. The Linker-Separated Subdomains of *E. coli* GntR and *B. subtilis* AraR, Formed High-Affinity D-Glucose Binding Pockets but Differed in Its Nonspecific Binding

The GntR repressor of *E. coli* regulates several operons involved in D-gluconate uptake and its catabolism via the Entner-Doudoroff pathway. Similarly, the AraR repressor controls arabinose metabolism in *B. subtilis*. Both TFs contain winged helix-turn-helix DNA-binding domains connected by linkers of 14–15 amino acids to C-terminal effector-binding domains. In both proteins, these effector domains consist of two subdomains separated by short linkers of 3–10 amino acids and each subdomain contains a central parallel β-sheet surrounded by four α-helices.

**Figure 6:**
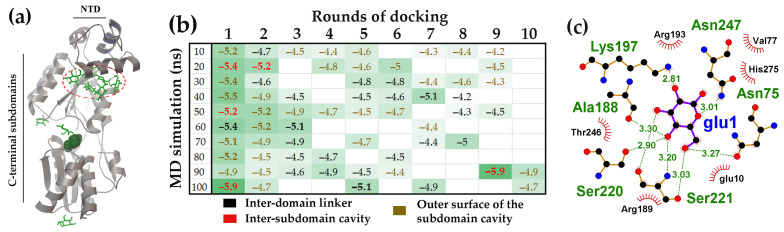
Accommodating D-glucose on its short inter-domain linker, GntR provides an inter-subdomain cavity in the CTD for high-affinity ligand interaction. (**a**) GntR monomer structures from a 100 ns MD trajectory with bound D-glucose molecules. The red oval outlines ligands that accumulate on the outer surface of the subdomain cavity. The ligand with the highest ΔG value is shown as a ball model, and the others as green rods. (**b**) Heat map representing the binding affinity (ΔG kcal/mol) of D-glucose for the 10–100 ns molecular models over successive molecular docking rounds (color code indicated). White cells correspond to random, nonspecific complexes on other protein surfaces, which had average ΔG value of −4.49 ± 0.20 kcal/mol. Ligands with ΔG > 3 StDs above the mean for nonspecific binding are shown in bold. (**c**) LigPlot+ diagram predicting the H-bond interactions for D-glucose from the first round of molecular docking.

Analysis of D-glucose interactions with GntR (Figure 6a; UniProt model P0ACP5) [87] revealed on average, only 2.3 D-glucose molecules interacted with the inter-domain linker of GntR (black ΔG values in Figure 6b). Nevertheless, the binding affinity in some models significantly exceeded the level of random attachment to the protein surface. The site with the highest affinity for D-glucose was found in the cavity formed between the two subdomains of the GntR CTD (Figure 6a).

The side chains of amino acid residues from the linkers connecting secondary structure elements are exposed within this cavity. Ligand binding to these residues bridges modules that are remote from each other in the protein primary sequence (Figure 6c). Although the cavity itself was relatively small and accommodated a maximum of two ligands (20 ns structure), an average of 3.5 D-glucose molecules were found attached to the inter-subdomain area outside the cavity (red oval in Figure 6a and brown ΔG values in Figure 6b). The observed affinity for D-glucose in this region may have evolved to capture and retain D-glucose, which, if shifted into the cavity, could potentially act as a structuring factor (Figure 6c).

**Figure 7:**
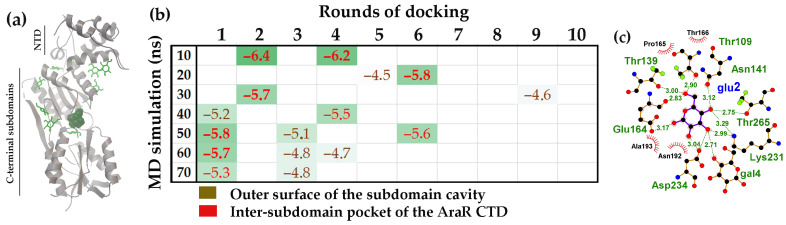
The inter-subdomain pocket of the AraR CTD exhibited the highest affinity for D-glucose. (**a**) AraR monomer structures from a 10 ns MD trajectory with bound D-glucose molecules. The ligand with the highest binding affinity is shown as a ball model; the other 9 molecules are displayed as green rods. (**b**) Heat map representing the binding affinity (ΔG, kcal/mol) of D-glucose for the 10–70 ns molecular models over successive molecular docking rounds (color code indicated). White cells correspond to nonspecific complexes on other protein surfaces, which had average ΔG value of −4.70 ± 0.31 kcal/mol. Ligands with binding efficiency greater than 3 StDs above the mean nonspecific binding are shown in bold. (**c**) LigPlot+ diagram predicting the H-bond interactions for D-glucose from the second round of molecular docking.

The effector-binding domain of the AraR protein is organized very similarly to that of GntR (Figure 7a). Although slightly larger (282 versus 267 residues), the CTD of AraR also consists of two subdomains that form a binding cavity where D-glucose bound to AraR with the highest affinity (Figure 7a,b). The amino acid residues forming hydrogen bonds with the ligand molecules within this cavity were located in unstructured regions that connect remote structural modules of the protein (Figure 7c). Furthermore, in six of the seven models, D-glucose was also found on the protein surface outside the inter-subdomain pocket (brown ΔG values in Figure 7b). However, unlike UxuR and GntR, the low-affinity complexes with D-glucose in AraR formed at sites that do not correspond to its unstructured linkers outside the main pocket (Figure 7b). This indicates that protein chain flexibility alone is insufficient to ensure carbohydrate binding.

It has long been known that the *B. subtilis* AraR protein has a “mosaic structure” combining the DNA-binding domain of the GntR family TFs with an effector-binding domain homologous to regulators of a GalR/LacI family [67,90]. This fundamental architectural difference could explain the dramatic contrast in how D-glucose interacted with the unstructured linkers of GntR and AraR outside their main binding pockets (Figure 6b and Figure 7b). However, a pairwise alignment of the E-domains from *E. coli* GntR and *B. subtilis* AraR, performed using the Needleman-Wunsch algorithm [91], unexpectedly revealed a higher degree of mutual homology between GntR of *E. coli* and AraR of *B. subtilis* (score 243.5) than between the GalR/LacI and AraR (scores 195.5 and 174.0, respectively). Given that the E-domains of both GntR and AraR have low homology scores (ranging from 11.0 to 44.5) to the CTDs of canonical GntR family members such as FarR (*E. coli*) and NagR (*B. subtilis*) [70], we propose that the *E. coli* GntR itself represents another example of a rare modular structure that combines functional domains from different evolutionary origins.

#### 2.2.2. The NagR and FarR Simulation Models Differed in the Structure of the Linker That Forms the High-Affinity Site in the CTD and in the Interaction Patterns with D-glucose

The NagR (YvoA) protein of *B. Subtilis* and the FarR (MngR) transcription factor of *E. coli* are both established members of the GntR family with a HutC-type effector-binding domain. The NagR repressor controls genes responsible for transporting and metabolizing the amino sugar N-acetylglucosamine (GlcNAc) [92]. Its activity is modulated by the effector molecules glucosamine-6-phosphate and/or N-acetylglucosamine-6-phosphate [93]. In contrast, the FarR (MngR) TF regulates only two genes involved in utilizing 2-O-α-mannosyl-D-glycerate (*mngA* and *mngB*) [94]; however, its potential effector has not yet been identified. Structurally, both proteins possess highly similar 70-residue wHTH DNA-binding domains (alignment score 133). They are built around a central β-sheet of six antiparallel strands, with four short α-helices formed by the linkers connecting the β-strands. At the domain junction, both proteins have a parallel β-sheet formed by the last β-strand from the NTD and a strand from the CTD tail.

**Figure 8:**
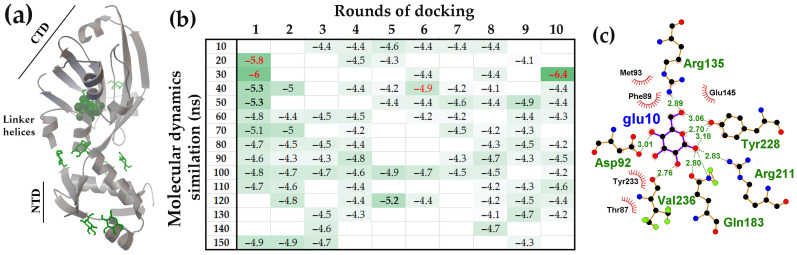
The pocket within the NagR CTD exhibited the highest affinity for interaction with D-glucose. (**a**) The NagR monomer structure from a 30 ns MD trajectory with bound D-glucose molecules. The ligand with the highest binding affinity is shown as ball model, and the other nine molecules are depicted as green rods. (**b**) Heat map representing the binding affinity (ΔG kcal/mol) of D-glucose for molecular models sampled over the 10–150 ns simulation across successive molecular docking rounds. ΔG values of ligands attached to flexible linkers are displayed in black, while those that entered the CTD pocket are highlighted in red. White cells correspond to nonspecific complexes on other protein surfaces, which had an average ΔG value of −4.46 ± 0.25 kcal/mol. Ligands with ΔG values greater than 3 StDs above the background are shown in bold. (**c**) LigPlot+ diagrams predicting H-bond formation for D-glucose from the tenth round of molecular docking.

The length of the polypeptide chains connecting these β-sheets to the central antiparallel sheet is also similar in both proteins (17 and 18 residues, respectively). Both linkers contain a short α-helix, which is two residues longer in NagR, spanning Phe89 to Ser95 (Figure 8a and PDB: 2WV0). Given the presence of multiple flexible elements in the NagR structure, such as loops between secondary structure modules and unstructured N- and/or C-terminal tails, it was not surprising to observe their frequent interaction with D-glucose (black ΔG values in Figure 8b). Over 10 rounds of molecular docking, we detected these regions occupied by an average of 5.8 ± 2.2 D-glucose molecules.

However, only three ligands, bound to the protein models from 40, 50 and 120 ns, of MD simulation exhibited the binding affinity for the linker regions significantly above the background level of −4.46 ± 0.25 kcal/mol. The preferred interaction site was a cavity bordered on one side by the central antiparallel β-sheet of the CTD and on the other by an unstructured polypeptide chain containing a short α-helix on the other (Figure 8a and red ΔG values in Figure 8b). This cavity was able to accommodate two ligand molecules (30 ns model). Predicted H-bonds were predominantly formed with residues of the β-sheet (Arg135, Gln183, Arg211, Tyr228 and Val236), but the positioning of D-glucose allowed for simultaneous interaction with Asp92 located on the short α-helix within the linker (Figure 8a,c). Of particular importance is the precise correspondence of this high-affinity D-glucose binding site to the X-ray-established effector-binding pocket [93], which accommodates glucosamine-6-phosphate and N-acetylglucosamine-6-phosphate via hydrogen bonds formed with Arg135, Gln183, and Tyr228 along with other residues like Thr87 and Phe89 from the flexible linker.

As in previous cases, models from different points in the MD trajectory varied in their ability to form complexes with D-glucose, both in interaction pattern and affinity. Surprisingly, however, for NagR, molecular docking revealed high-affinity D-glucose binding at the effector site only in models from the early stages of the MD simulation (red ΔG values in Figure 8b). Extending the simulation beyond 100 ns we revealed no trend toward increased D-glucose binding at this site (Figure 8b). If the dynamic simulation reflects stabilization into a conformation specific for binding the natural inducers, this may indicate that protein maturation reduces the accessibility of the effector site to a nonspecific ligand. In NagR, such a “stabilization” could have occurred at approximately 38 ns of the MD trajectory when a 12 ns plateau was followed by a sharp RMSD decrease, after which further structural adaptation proceeded (Supplementary Figure S1).

**Figure 9:**
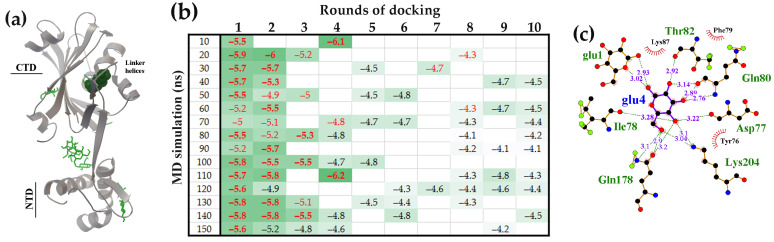
The pocket within the FarR CTD, bordered by inter-domain linker, exhibited the highest affinity for D-glucose. (**a**) The FarR monomer structure from a 10 ns MD trajectory with bound D-glucose molecules. The ligand bound to the high-affinity site is shown as ball model. (**b**) Heat map depicting the binding affinity (ΔG kcal/mol) of D-glucose for molecules obtained from 10 to 150 ns simulations in successive rounds of molecular docking. ΔG values of ligands attached to linkers are shown in black, while those that entered the high-affinity pocket are displayed in red. Nonspecific complexes on other protein surfaces had an average ΔG value of −4.53 ± 0.26 kcal/mol. Ligands ΔG values greater than 3 StDs above the background are shown in bold. (**c**) LigPlot+ diagrams showing the H-bonds potentially formed by D-glucose from the 4th round of docking.

Although the FarR protein of *E. coli* has a structural organization highly similar to that of NagR (Figure 9a and P13669 model in UniProt), its interaction pattern with D-glucose was significantly different (Figure 9b).

The primary distinction was a markedly higher level of D-glucose binding at the potential ligand-binding pocket. Over 10 rounds of molecular docking, this pocket sequestered an average 2.60 ± 0.83 D-glucose molecules (Figure 9b), while only 2.53 ± 1.55 ligands interacted with unstructured linkers in other protein regions (black ΔG values in Figure 9b). Unlike NagR, all structural models of FarR recruited D-glucose to this pocket (red ΔG values in Figure 9b) and the amino acid residues within this pocket that formed H-bonds with D-glucose were predominantly from the flexible linker (Asp77, Ile78, Gln80, Thr82) (Figure 9c). The high-affinity cavity was sufficiently large to accommodate up to four D-glucose molecules (20 ns model), which often formed H-bonds with each other. Similarly to the NagR effector-binding site, the D-glucose attached to the flexible linker of FarR simultaneously interacted with Lys204 and/or Gln178 from two β-strands (Figure 9c). This interaction could mediate conformational transitions affecting the relative orientation of the N-terminal and C-terminal domains. The distinct D-glucose interaction patterns observed for FarR and NagR suggest that FarR may be specifically sensitive to the presence of D-glucose.

#### 2.2.3. A Putative Transcription Factor YydK with Structural Homology to NagR and FarR Interacted with D-Glucose in a NagR-like Manner

**Figure 10:**
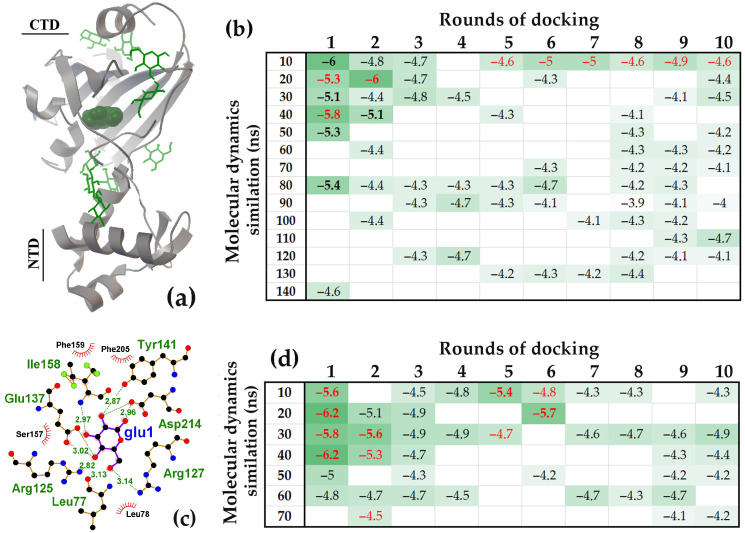
A putative transcription factor YydK, with structural and sequence homology to NagR and FarR, interacted with D-glucose in a NagR-like manner. (**a**) The YydK monomer structure from a 40 ns MD trajectory (**d**) with bound D-glucose molecules. The ligand bound to the high-affinity site is shown as ball model. (**b**,**d**) Heat maps representing the binding affinity (ΔG kcal/mol) of D-glucose for molecular models from different time points of the MD simulation trajectory across successive molecular docking rounds. ΔG values of ligands attached to flexible linkers are shown in black, while those that entered the high-affinity pocket are displayed in red. Nonspecific complexes on other protein surfaces had an average ΔG value of −4.45 ± 0.22 (**b**) and −4.53 ± 0.29 kcal/mol (**d**). Ligands with a binding efficiency greater than 3 StDs above the background are shown in bold. (**c**) LigPlot+ diagram predicting the H-bonds formation for D-glucose from the first round of molecular docking (40 ns MD trajectory, **d**).

The YydK protein from *B. subtilis* in our dataset represents an uncharacterized transcription factor with a known X-ray structure (PDB: 3bwg). We selected YydK for analysis due to its close structural (Figure 10a) and sequence homology with both NagR and FarR. Similarly to these proteins, the winged helix-turn-helix N-terminal domain of YydK is connected by a 15 residue linker (Leu77–Asn91) to a HutC-type CTD containing a six-stranded antiparallel β-sheet and four α-helices. This linker is unstructured in the YydK model of UniProt (ID Q45591), in the crystal structure (PDB: 3bwg), and was truncated in our molecular models (Figure 10a).

Over 10 rounds of molecular docking, the flexible linkers outside the potential effector-binding site were occupied by an average of 4.21 ± 1.96 D-glucose molecules (black ΔG values, Figure 10b). This occupancy is intermediate between that of NagR (5.8 ± 2.2) and FarR (2.53 ± 1.55). However, in contrast to FarR but similar to NagR, the CTD pocket of YydK sequestered D-glucose in only three of its structural models (red ΔG values, Figure 10b). The amino acid residues within this pocket that interacted with D-glucose were predominantly located on β-sheet strands (Arg125, Arg127, Glu137, Tyr140, Ile158 and Asp117) (Figure 10c). The interaction pattern, characterized by only a single bridging H- bond with the linker residue Leu77, was also observed in NagR (Figure 10c). To verify the lower occupancy of the YydK pocket compared to FarR, we repeated the molecular docking using structural models from an independent 70 ns simulation. Notable, two RMSD profiles obtained from independent MD simulations revealed a prolonged plateau that began at approximately 30 ns (Supplementary Figure S1). As expected, the external linker regions in these new models adopted different, dynamically variable conformations, which altered the surface distribution of D-glucose (Figure 10b,d). However, the pattern of D-glucose interaction with the effector-binding site remained similar to that of NagR (Figure 8a).

Thus, while the inter-domain linker in all FarR models actively participated in virtual ligand binding (primarily in the initial docking rounds), similarly positioned linkers in NagR and YydK exhibited this capability only during the early stages of MD simulation. A key distinction in these interactions is the location of hydrogen bonding: in NagR and YydK, bonds were preferentially formed with structured domain regions, primarily β-sheets, whereas in FarR, most bonds were formed with amino acids on the inter-domain linker itself.

## 3. Discussion

The study of proteins with intrinsically disordered regions (IDRs) is an emerging frontier in protein science and molecular biology. Although existing research is predominantly focused on eukaryotes [9,95,96], bioinformatic studies have identified IDRs across various functional classes of prokaryotic proteins [58]. This includes transcription regulators, for which the occurrence of disordered regions was found to be explicitly lower compared to eukaryotic regulators [18,96]. This discrepancy was reasonably explained by the greater complexity of transcriptional regulation in higher organisms. Nevertheless, growing evidence demonstrates that IDRs are functional in bacterial regulators [7,8,97]. This study addressed a key aspect of that functionality: the accessibility of IDRs in bacterial TF to regulatory signals from potential effector molecules.

Molecular dynamics simulations [75] were employed to assess the plasticity of unstructured linkers and to generate conformational ensembles for molecular docking. In the UxuR monomer, the inter-domain linker underwent rearrangements characterized by the transient formation and dissolution of short α-helix and β-strand elements (Figure 1b). A similar variability in β-strand formation was also observed in the UxuR dimer, while the α-helix formed only once (in the 100 ns model). When considering this variance as a form of virtual structural sampling, we found no correlation between the presence of these transient structured modules and either linker occupancy by carbohydrates or their binding affinity. We did not detect extensive interactions between D-glucose and the AraR inter-subdomain linkers, which remained unstructured in all models. Moreover, among the three structurally similar proteins (FarR, NagR, and YydK), only the inter-domain linker of FarR exhibited preferential and reproducible formation of statistically significant complexes with D-glucose. Therefore, the mere presence of an unstructured region in the polypeptide chain is an insufficient criterion to predict or exclude its capacity for ligand binding.

Nevertheless, for every protein examined, molecular docking revealed conformations of the weakly structured or unstructured regions that were able to form complexes with binding affinities comparable to those of specific carbohydrate interactions at the effector-binding sites. The affinity of D-galacturonate for the UxuR inter-domain linker (−6.8 kcal/mol) was even higher than that of its natural ligands, D-fructuronate and D-glucuronate, for the effector-binding pocket of the UxuR dimer (−6.7 ≤ ΔG ≤ −5.4 kcal/mol). In AraR and GntR, the highest-affinity complexes with D-glucose (−6.4 kcal/mol) were localized in their inter-subdomain spaces, which consist primarily of short 3-10-residue linkers connecting the protein’s structural modules. According to Protein-Ligand Interaction Profiler (PLIP) analysis [77], D-glucose molecules can form seven hydrogen bonds within the AraR CTD pocket, all exclusively with residues from four different linkers. In GntR, only one of the seven H-bonds predicted for the preferred ligand involved the α-helix residue (Lys197), while the rest were suggested for interaction with unstructured regions of the protein chain. Given that all tested carbohydrates can form extensive hydrogen bond networks with polar/charged residues or the protein backbone, their interactions with linkers may serve as an important structuring factor that induces or modulates functional conformations.

Typically, the transient conformations of flexible protein regions do not allow a ligand to form a sufficiently stable complex. Certain conditions, like dynamic conformational adaptation or initial low-affinity ligand interaction, are often required to achieve a binding-competent state. It is therefore surprising that, in the UxuR monomer, even low-affinity ligand interactions can promote the formation of extended clusters at the dimerization interface. The absence of such aggregates in the dimer suggests that they may hinder UxuR maturation, i.e., have a functional purpose. This raised the question of whether H-bonding between several ligands, accidentally positioned in close proximity on the protein surface, could contribute to stochastic cluster formation. In other words, could multi-ligand docking generate artefactual results that reflect not the properties of the flexible linker but the binding affinity of the ligands to each other?

**Figure 11:**
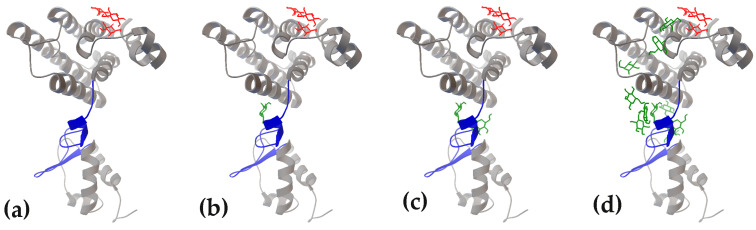
Virtual experiment validating the selective, low-affinity interaction of D-glucose with the UxuR monomer inter-domain linker. (**a**) UxuR monomer complex with three D-glucose molecules that was used as the input for the subsequent docking of one (**b**), two (**c**) and ten (**d**) rounds.

To test this assumption, we used an UxuR monomer model taken from a 50 ns MD trajectory, accommodated nine D-glucose molecules in the course of sequential molecular docking (Figure 1c). Those included one molecule that had bound nonspecifically to the surface of the CTD in the fourth docking round, and two additional D-glucose molecules that formed H-bonds with the first one in the fifth and ninth rounds. Ligands bound by UxuR in rounds 1–3 and 6–8 were removed from the complex. The resulting complex (Figure 11a) was then subjected to docking with ten new D-glucose molecules (Figure 11b–d).

As expected, most of them, including the first two (Figure 11b–d), bound to the inter-domain linker, exhibiting the same low affinity as observed in the original experiment (Figure 1c). Several D-glucose molecules were distributed over the protein surface, but none of them joined to the pre-placed cluster. Thus, sequential molecular docking of carbohydrates onto the metabolic regulator UxuR appears to reveal a specific property of its flexible inter-domain linker: the ability to accumulate carbohydrates, each engaged only in weak nonspecific interactions. These results lead us to a hypothesis that unstructured linkers in UxuR may function as sensors of intracellular concentrations of substrates and intermediates from central metabolic pathways.

It is unsurprising that of the 33 hydrogen bonds predicted by the PLIP algorithm in the highest-affinity carbohydrate complexes with unstructured linkers, 26 involved optimal H-bond formers: asparagine, serine, aspartic acid and threonine. In contrast, H-bonds with glutamine and glutamic acid within high-affinity pockets were primarily associated with α-helices and β-strands. In unstructured regions only three H-bonds involved glutamine, and one was formed with glutamic acid. This asymmetry between Asn/Asp and Gln/Glu suggests a certain binding “specificity” and corroborates the underrepresentation of glutamine (and, to a lesser extent, glutamic acid) in small-molecule metabolite binding to 45 *E. coli* transcription factors [98]. Given that Gln/Glu possess longer side chains than Asn/Asp, these observations suggest that successful ligands, which successively bind within unstructured regions, may encounter a size constraint, likely mirroring a geometric selectivity inherent to natural effector binding sites.

In our docking experiments, where four ligand types were screened against UxuR structures corresponding to different points of the MD trajectory, we did not observe statistically significant differences in their binding affinities. This suggest a biological model in which the four carbohydrates compete for the linker conformations that are most favorable for high-affinity binding. Consequently, a regulatory circuit may exist where protein activity is controlled by the relative concentrations of these carbohydrates. For example, a high intracellular concentration of D-glucose, which is not the primary effector for UxuR, could allow it to bind not only to the inter-domain linkers but also to compete with the primary effectors, D-fructuronate and D-glucuronate, for the effector site in the C-terminal domain. This would enable glucose to suppress the activation of the Ashwell and Entner-Doudoroff pathways even when primary effectors are abundant. Such a mechanism could represent a significant component of the catabolite repression orchestrated in response to variations in D-glucose concentration.

Extending this regulatory principle to other bacterial proteins with unstructured ligand-binding regions suggests the existence of overlapping cellular regulatory networks sensitive to the concentration of key metabolites. The formation of low-affinity complexes at transient interaction sites can maintain a baseline protein activity, while inherent conformational flexibility enables rapid activation upon the arrival of a specific effector. The effectiveness of such networks likely derives from fundamental biophysical properties of the protein chain, minimizing the need for precise evolutionary adaptation to individual ligands.

## 4. Materials and Methods

### 4.1. Structural Models of Carbohydrates Used in the Study

The three-dimensional structural models of the ligands (D-glucose, D-fructuronate, D-galacturonate and D-glucuronate) were obtained from the PubChem database [99]. They were prepared for molecular modeling using Avogadro (v. 1.2.0.) [100].

### 4.2. Structural Models of Transcription Factors Used in the Study

The UxuR protein sequence from *E. coli* K-12 MG1655 (NC_000913) [101] was obtained from the KEGG [102] (entry T00007, K13637). It was selected due to its partially characterized ligand-binding properties [73,80,82,83]. Both monomeric and dimeric three-dimensional structures of UxuR were modeled using the crystal structure of the GntR protein from *Streptococcus agalactiae* (PDB: 6AZ6) as a template. To select additional transcription factors for analysis, we retrieved the amino acid sequences of 27 candidate proteins from *E. coli* and *B. subtilis* using KEGG database [102]. A three-dimensional structure for each candidate was generated using the SWISS-MODEL online server [85,86]. Five TFs were subsequently chosen based on two criteria: (1) the presence of unstructured inter-domain linkers, a hallmark of GntR-family regulators, and (2) the quality and completeness of the predicted models, requiring no large sequence breaks or major deletions. The selected set included GntR and FarR (MngR) from *E. coli* K-12 MG1655 (NC_000913) [103], NagR (YvoA) and YydK from *B. subtilis* subsp. *subtilis* str. 168 (NC_000964.3) [97] and the AraR protein from *B. subtilis* strain FX-1 (CP004019) [104].

Among the selected transcription factors, only NagR and YydK have experimentally determined crystal structures of their apo-forms, with resolutions of 2.051 Å (PDB: 2WV0) and 2.09 Å resolution (PDB: 3BWG), respectively. However, the YydK structure contains an engineered expression tag at its N-terminus. NagR has also been co-crystalized with its effector molecules, N-acetylglucosamine-6-phosphate (PDB: 4U0W, 2.001 Å) and glucosamine-6-phosphate (PDB: 4U0V, 2.051 Å). The availability of these ligand-bound structures allowed us to compare our molecular docking results with the natural effector-binding pocket. For GntR, crystal structures are available from three different organisms, though each has limitations. Structures from *Thermotoga maritima* (PDB: 3FMS, 2.2 Å) [105] and *Chromobacterium violacium* (PDB: 3H5O, 2.3 Å) contain engineered expression tags, and the 3FMS structure also bears three point substitutions. A structure of a putative GntR from *Rhodococcus* species RHA1, crystallized from native protein (2.2 Å), possesses only two acetate molecules in its effector-binding site but features an antiparallel β-sheet within its 16-residue inter-domain linker. For FarR and AraR only AlphaFold-predicted models are available (UniProt entries P13669 and P96711, respectively) [61]. Therefore, to minimize potential artifacts from the heterogeneous experimental conditions and to ensure a consistent structural alignment across all models, we used predicted models for all proteins in this study. The structures of GntR and FarR from *E. coli* K-12 MG1655 (NC_000913) were modeled using the SWISS model P0ACP6 and PDB template 4U0W, respectively. NagR and YydK from *B. subtilis* subsp. *subtilis* str. 168 (NC_000964.3) were modeled using PDB templates 2WV0 and 3BWG, respectively. The AraR protein from *B. subtilis* strain FX-1 (CP004019) was obtained based on the AlphaFold [61] model D4G1L9 as a template.

### 4.3. Molecular Dynamic Simulation

To assess atomic positional fluctuations and to select conformers for docking, the obtained structures were subjected to MD simulations using the Open MMZephyr software package (v. 2.0.3) [106]. Simulations were performed at 310.15 K using the Amber96 force field and an explicit solvent model (“accurate water”). MD simulations for the UxuR monomer and dimer were carried out independently. A total of 78 structural models, comprising 6 monomers and the UxuR dimer, were collected from subsequent time points along the MD trajectories. For molecular docking, structures were extracted at 10 ns intervals from the pre-optimized time range for each protein (maximal range: 10–150 ns). This sampling strategy enabled the visualization of the flexibility in the proteins’ unstructured linkers and allowed for comparison of ligand binding patterns across the simulation trajectory. To assess the stability and convergence of the MD simulations, root mean square deviation (RMSD) profiles were generated for each trajectory over the simulation time. This analysis was performed using Visual Molecular Dynamics (VMD) software, version 1.9.1 [107]. RMSD profiles for all studied proteins are provided in Supplementary Figure S1.

### 4.4. Flexible Molecular Docking

The use of sequential flexible molecular docking is motivated by its ability to predict low-affinity ligand-target interactions. It was performed using the AutoDock Vina software package (v. 1.1.2) [76]. All rotatable bonds in the ligands were treated as flexible. For each ligand, the preferred binding sites were identified, and the corresponding binding affinity (ΔG in kcal/mol) was calculated using the explicit solvent model “accurate water”. A sequential docking protocol was employed for all ligands assessed and all 78 protein models. In each subsequent round, the ligand with the highest affinity from the previous round was added to the target protein structure, and the resulting complex was used as the new input for the next docking round. This iterative process was repeated ten times. The specificity of ligand binding was evaluated based on two parameters: (1) the calculated binding affinity (ΔG) with unstructured polypeptide chains and (2) the difference in ΔG from ligands binding nonspecifically to other protein surfaces. Docking results were visualized using AutoDockTools (v. 1.5.6) [108]. Potential hydrogen bonds in the protein-ligand complexes were predicted and visualized using LigPlot+ (v. 2.2.8) [77]. The same as any experimental tool, sequential docking has its limitations, particularly the need to select an optimal number of iterations: enough to yield a clarifying result, but not so many as to produce noisy data from excessive docking rounds. In our view, using 10 docking iterations strikes a reasonable compromise between these competing requirements.

### 4.5. Statistics

To assess the statistical significance of carbohydrate interactions with unstructured regions in the studied TF, we calculated the average affinity of nonspecific ligand binding with protein models outside the linker regions and effector-binding pockets. The binding affinities obtained from protein conformers at different simulation time points were pooled for each ligand. This yielded between 17 and 26 ΔG values for the UxuR monomer and dimer (5 time points). The number of glucose molecules involved in nonspecific interactions with the other transcription factors ranged from 36 (GntR, 10 time points) to 74 (YydK, 14 time points). These data provided a baseline interaction level for all protein-carbohydrate pairs, enabling identification of complexes with binding affinities more than 3 standard deviations above this mean (*p*-value < 0.05). To assess differences in the binding efficiencies of the four ligands to the UxuR monomer and dimer, we used SigmaPlot (v11.0). Pooled sets of affinity values, obtained at different time points along the MD trajectory for each carbohydrate, were tested for normality using the Shapiro–Wilk test. In almost all cases, the datasets violated this assumption. Therefore, affinity comparisons were performed using the nonparametric Mann–Whitney U test (using SigmaPlot’s “Compare Two Groups” function). Even without applying corrections for multiple comparisons, we found no statistically significant differences in the binding efficiencies of the four ligands.

Pairwise protein sequence alignments were performed using the Needleman-Wunsch algorithm [91] implemented in the European Molecular Biology Open Software Suite (EMBOSS).

## Data Availability

The original contributions presented in this study are included in the article/Supplementary Material. Further inquiries can be directed to the corresponding authors.

## Supplementary Materials

The following supporting information can be downloaded at: https://www.mdpi.com/article/10.3390/ijms27020941/s1.
