# Supplementary material for: Molecular Strategies of Carbohydrate Binding to Intrinsically Disordered Regions in Bacterial Transcription Factors
Source: Int J Mol Sci. 2026 Jan 17;27(2):941. doi: 10.3390/ijms27020941 (PMC12842492; doi:10.3390/ijms27020941)
Supplement: Supplementary file 1 [file ijms-27-00941-s001.zip › ijms-4012991-supplementary.pdf]

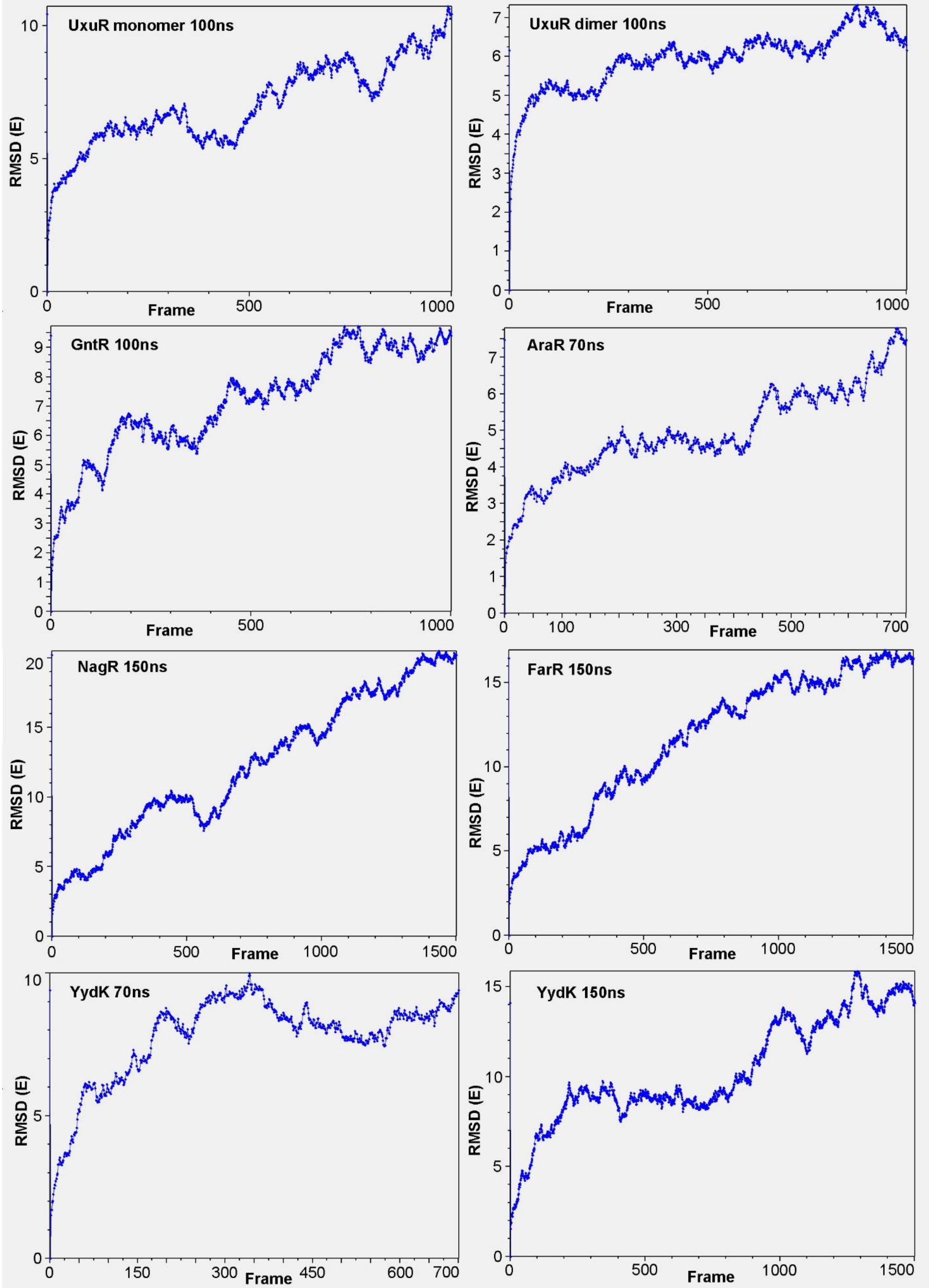

Supplementary Figure S1. Root mean square deviation profiles calculated over the Molecular Dynamics simulation time (0–150 ns).
